# Glutathionylation of the Bacterial Hsp70 Chaperone DnaK Provides a Link between Oxidative Stress and the Heat Shock Response*

**DOI:** 10.1074/jbc.M115.673608

**Published:** 2016-01-28

**Authors:** Hong Zhang, Jie Yang, Si Wu, Weibin Gong, Chang Chen, Sarah Perrett

**Affiliations:** From the ‡ National Laboratory of Biomacromolecules, Institute of Biophysics, Chinese Academy of Sciences, 15 Datun Road, Chaoyang District, Beijing 100101, China,; § University of the Chinese Academy of Sciences, 19 Yuquan Road, Shijingshan District, Beijing 100049, China, and; ¶ Beijing Institute for Brain Disorders, Beijing 100069, China

**Keywords:** 70 kilodalton heat shock protein (Hsp70), chaperone, chaperone DnaK (DnaK), glutathionylation, oxidative stress, S-glutathionylation, deglutathionylation, thiol modification

## Abstract

DnaK is the major bacterial Hsp70, participating in DNA replication, protein folding, and the stress response. DnaK cooperates with the Hsp40 co-chaperone DnaJ and the nucleotide exchange factor GrpE. Under non-stress conditions, DnaK binds to the heat shock transcription factor σ^32^ and facilitates its degradation. Oxidative stress results in temporary inactivation of DnaK due to depletion of cellular ATP and thiol modifications such as glutathionylation until normal cellular ATP levels and a reducing environment are restored. However, the biological significance of DnaK glutathionylation remains unknown, and the mechanisms by which glutathionylation may regulate the activity of DnaK are also unclear. We investigated the conditions under which *Escherichia coli* DnaK undergoes *S*-glutathionylation. We observed glutathionylation of DnaK in lysates of *E. coli* cells that had been subjected to oxidative stress. We also obtained homogeneously glutathionylated DnaK using purified DnaK in the apo state. We found that glutathionylation of DnaK reversibly changes the secondary structure and tertiary conformation, leading to reduced nucleotide and peptide binding ability. The chaperone activity of DnaK was reversibly down-regulated by glutathionylation, accompanying the structural changes. We found that interaction of DnaK with DnaJ, GrpE, or σ^32^ becomes weaker when DnaK is glutathionylated, and the interaction is restored upon deglutathionylation. This study confirms that glutathionylation down-regulates the functions of DnaK under oxidizing conditions, and this down-regulation may facilitate release of σ^32^ from its interaction with DnaK, thus triggering the heat shock response. Such a mechanism provides a link between oxidative stress and the heat shock response in bacteria.

## Introduction

Hsp70 is conserved in Archaea, Bacteria, and Eukarya and is essential for survival (1). DnaK is the major bacterial Hsp70. Its structure contains two domains: the ATPase or nucleotide-binding domain (NBD)^3^ and the substrate-binding domain (SBD). The SBD is composed of an all β-sheet subdomain (SBDβ), which provides the substrate binding cavity, and a C-terminal α-helical lid subdomain (SBDα) (2–4). Allosteric conformational changes in Hsp70 couple the ATP hydrolysis cycle in the NBD and the substrate binding/release cycle in the SBD. In the ATP-bound state, substrate binds to the SBD in its SBDα lid open state by relatively weak interactions that can promote ATP hydrolysis in the NBD (3–8). After ATP hydrolysis, the NBD is in the ADP-bound state, leading to strong interaction between substrate and the SBD in its SBDα lid closed state (2, 5–9). Nucleotide exchange factors promote exchange of ADP with ATP in the NBD, which then causes loosening of the interaction between substrate and the SBD and facilitates substrate release and exchange (10–12). NMR and EPR studies have revealed that in the ATP-bound state the NBD and SBD of DnaK are in a docked position, and the lid is in an open position; whereas in the ADP-bound or apo state, the NBD and SBD of DnaK are undocked, and the lid is predominantly in a closed position. Peptide binding to the ATP-bound state of DnaK leads to undocking of the NBD and SBD (5, 13).

It has been found that the functional cycle of Hsp70 can be regulated by a series of factors, including Hsp40, nucleotide exchange factors, and tetratricopeptide repeat-containing proteins (14, 15). DnaJ is the Hsp40 partner of DnaK and interacts with the NBD and the SBD, promoting both ATPase activity and substrate binding of DnaK (16, 17). GrpE is the nucleotide exchange factor for DnaK and interacts with the NBD of DnaK with high affinity to facilitate ADP/ATP exchange (11, 18). The combination of DnaJ and GrpE markedly accelerates the functional cycle of DnaK in *Escherichia coli* (10, 19).

The DnaK-DnaJ-GrpE complex works as a chaperone machine to mitigate the effects of heat shock stress, prevent protein aggregation, and facilitate protein folding (20). This machinery also controls the level of the heat shock response by interaction with the σ^32^ subunit of RNA polymerase, which is involved in expression of a number of heat shock-related genes (21, 22). Under non-stress conditions, formation of the DnaK-DnaJ-GrpE-σ^32^ complex inactivates σ^32^ and facilitates its degradation by the protease FtsH (20, 23, 24). Stress causes σ^32^ to be released from the DnaK-DnaJ-GrpE-σ^32^ complex, which triggers expression of heat shock genes. Thus σ^32^ plays a similar role to the transcription factor Hsf1 in eukaryotes (25, 26). However, the mechanism by which σ^32^ (or Hsf1) is rapidly released from the Hsp70 complex remains unclear.

Bacteria often encounter oxidative stress when they are attacked by reactive oxygen and reactive chlorine species released from the immune system of the host. It is therefore interesting to note that DnaK becomes inactive under conditions where oxidative stress is accompanied by heat shock (27). The following factors are likely to contribute to the mechanism of inactivation of DnaK. (i) The dramatic decrease in cellular ATP levels due to oxidative stress will limit the function of ATP-dependent chaperones. (ii) Depletion of ATP and elevated temperature will lead to unfolding of the NBD of DnaK. (iii) Unfolding of the NBD of DnaK leaves its cysteine residue vulnerable to thiol modification (27). Reversible thiol modifications such as glutathionylation will cause DnaK to remain inactivated until both cellular ATP levels and reducing conditions are restored (27). A number of cysteine modifications of DnaK have been observed *in vivo* under oxidative stress conditions, including *S*-nitrosylation (28) as well as sulfinic, sulfenic, and sulfonic forms (27), indicating that the cysteine residue of DnaK is indeed susceptible to modification under these conditions.

Oxidative stress can induce heat shock gene expression and the heat shock response, similar to heat stress in bacteria and eukaryotes (29, 30). It is reported that under oxidative stress conditions the half-life of σ^32^ becomes longer due to its release from the chaperone complex (31). There is still no convincing explanation for the link between oxidative stress and release of σ^32^ from DnaK. However, the observation of cysteine modifications such as glutathionylation under oxidative stress conditions could provide a clue to this mystery.

To address the significance of DnaK glutathionylation and the mechanism by which glutathionylation regulates the function of DnaK, we investigated the conditions under which the cysteine residue of DnaK becomes glutathionylated and then studied the effect of glutathionylation on the structure and function of DnaK. We found that glutathionylation affects the interaction of DnaK with its key partners DnaJ, GrpE, and σ^32^. This study indicates a mechanism by which DnaK is able to transfer redox information and thus could provide a bridge between oxidative stress and the heat shock response.

## Experimental Procedures

### 

#### 

##### Protein Expression and Purification

The genes for *dnaK*, *dnaJ*, *grpE*, *rpoH* (for production of σ^32^), and *grxC* (for production of Grx3) were cloned from the genomic DNA of *E. coli* C41 cells. The *dnaK* truncations were derived from the wild-type *dnaK* gene. *dnaK*, its truncations, *rpoH*, and *grxC* genes were ligated into the pET28a-*SMT3* expression plasmid for production of protein with a cleavable His_6_-Smt3 tag. *dnaJ* and *grpE* genes were ligated into the mini pRSETa expression plasmid for production of protein with an uncleavable His_6_ tag. Primer sequences are shown in Table 1. All the constructs were confirmed by DNA sequencing.

**TABLE 1:** **Primers used for cloning and mutation of proteins used in this study** The underlined residues indicate start and stop codons.

Protein name	Primers
WT DnaK	Forward, 5′-CATCACGGATCCATGGGTAAAATAATTGGTATCGACCTGGGTA-3′
	Reverse, 5′-CGCTCGAAGCTTTTATTTTTTGTCTTTGACTTCTTCAAATTCAGCG-3′
Δ508–638DnaK	Forward, 5′-CATCACGGATCCATGGGTAAAATAATTGGTATCGACCTGGGTA-3′
	Reverse, ′-CGCTCGAAGCTTACAGACCAGAAGAAGCCTTGATGGTGAT-3′
Δ553–638DnaK	Forward, 5′-CATCACGGATCCATGGGTAAAATAATTGGTATCGACCTGGGTA-3′
	Reverse, 5′-CGCTCG AAGCTTATTCTTCAACCTGCTTACGGGTGCTGTGC-3′
Δ606–638DnaK	Forward, 5′-CATCACGGATCCATGGGTAAAATAATTGGTATCGACCTGGGTA-3′
	Reverse, 5′-CGCTCGAAGCTTATTGCTGCTGGGCGATTTCCATCAGTTT-3′
DnaK T199A	Forward, 5′-GTGGTGCTTTCGATATTTCTATTATCGAAATCGACG-3′
	Reverse, 5′-CGAAAGCACCACCACCCAGGTCATAAACCGC-3′
Grx3	Forward, 5′-CATCACGGATCCATGGCCAATGTTGAAATCTATACC-3′
	Reverse, 5′-CGCTCGAAGCTTATTTCAGCAGGGGGTCCAGTCCACCAC-3′
DnaJ	Forward, 5′-GGGCACGGATCCAAGATGGCTAAGCAAGATTATTACG-3′
	Reverse, 5′-ACACTTCCATGGTTAGCGGGTCAGGTCGTCAAAAA-3′
GrpE	Forward, 5′-AACTGGCTCGAGATGAGTAGTAAAGAACAGAAAACGC-3′
	Reverse, 5′-ACACCTCCATGGTTAAGCTTTTGCTTTCGCTACAGTA-3′
σ^32^	Forward, 5′-GAGAGGGGATCCATGACTGACAAAATGCAAAGTTTAGC-3′
	Reverse, 5′-ATAGCGAAGCTTACGCTTCAATGGCAGCACGCAAT-3′

All the proteins were produced in BL21(DE3) *E. coli* cells, induced with 0.2 mm isopropyl 1-thio-β-d-galactopyranoside, and grown at 16 °C for 16–20 h. The harvested cells were lysed using a JNBIO JN-3000 PLUS high-pressure cell press in Buffer A (50 mm Tris-HCl buffer, pH 7.5, containing 300 mm NaCl) containing 10 mm imidazole and 2 mm β-mercaptoethanol, and the debris was removed by centrifugation (35,000 × *g*, 45 min). The supernatant was then loaded onto a nickel affinity column (chelating Sepharose Fast Flow resin, GE Healthcare) and washed with Buffer A containing 50 mm imidazole. Proteins were eluted using Buffer A containing 200 mm imidazole. His_6_-Smt3-DnaK, His_6_-Smt3-fused DnaK truncations, and His_6_-Smt3-Grx3 were cleaved by the protease Ulp1 at 4 °C for 1 h to remove the His_6_-Smt3 fusion tag. Then the cleaved proteins were loaded onto the nickel affinity column again after changing to Buffer A containing 10 mm imidazole, and the run-through was collected for further SEC purification. The concentrated proteins were loaded onto a 120-ml Superdex^TM^ 200 HiLoad column (GE Healthcare) equilibrated with Buffer B (50 mm Tris-HCl buffer, pH 7.5, containing 100 mm KCl and 5 mm MgCl_2_). The dimer and monomer peaks were collected for glutathionylation experiments. His_6_-DnaJ, His_6_-GrpE, and His_6_-Smt3-σ^32^ were further purified using a 120-ml Superdex^TM^ 200 HiLoad column equilibrated with Buffer B. All protein concentrations are given in terms of monomer and were determined by a bicinchoninic acid (BCA) assay kit (Pierce).

##### Measurement of Cysteine Reactivity in DnaK

Cysteine reactivity of DnaK was measured by Ellman assay as described (32) to predict the possibility of glutathionylation. A standard curve was obtained using 0–100 μm free cysteine. We mixed 5 μl of 10 mm DTNB in 50 mm Na_2_HPO_4_/NaH_2_PO_4_ buffer, pH 7.5, with 145 μl of 10–20 μm DnaK, and the absorbance at 412 nm was measured in a SpectraMax M3e multimode plate reader (Molecular Devices) at room temperature. If ADP, ATP, or peptide was added, 1 mm ADP/ATP/NRLLLTG peptide was mixed with DnaK to give a total volume of 145 μl, and the mixture was allowed to stand at room temperature for 1 h before DTNB was added. The number of active cysteine residues can be calculated by dividing the concentration of free thiols in the protein by the concentration of protein.

To measure the p*K_a_* of the cysteine residue in DnaK, we recorded the specific absorbance of the thiolate anion at 240 nm according to the pH-dependent ionization of the nucleophilic cysteine (33). Measurements using 15 μm DnaK were carried out at 25 °C in Buffer B in a 1-cm-path length, 0.4-ml-volume cuvette on a Shimadzu UVmini-1240 instrument. The pH of the protein solution was increased from 7.5 to 12.5 by the stepwise addition of calculated volumes of 6 n KOH in Buffer B. Absorption of buffer at different pH values was subtracted. Molar absorbance was corrected according to the actual volume and concentration of DnaK. The p*K_a_* value was estimated from the curve of the pH-dependent absorption at 240 nm according to the Henderson-Hasselbalch equation. The predicted p*K_a_* value was calculated using online Propka software using the NMR structure of DnaK (Protein Data Bank code 2kho). For the DnaK homologue of *Salmonella enterica* serovar Typhimurium, the structure was modeled using the software Modeller (34) using the *S. enterica* Typhimurium DnaK sequence and the *E. coli* DnaK structure (Protein Data Bank code 2kho). The predicted p*K_a_* value was then calculated as above.

##### Preparation of Glutathionylated and Deglutathionylated DnaK

A solution of 15 μm DnaK or its mutant was incubated in the dark at 37 °C for 1 h in the presence of 1 mm GSH and 1 mm diamide to allow the glutathionylation reaction to proceed. To deglutathionylate the protein, 10 mm DTT was added to the reaction mixture. GSH, diamide, and/or DTT was removed by dialysis. (To prevent oxidation of the untreated DnaK control during dialysis, 10 mm DTT was added before dialysis.) To test the effect of nucleotide on glutathionylation of DnaK, 0.5 mm ATP or ADP was premixed with DnaK T199A for 30 min at room temperature before GSH and diamide were added. To test deglutathionylation of DnaK by Grx3, 10 μm glutathionylated DnaK was incubated for 30 min at 37 °C under each of the following conditions: 20 μm GSH, 40 μm GSH, 1.5 μm Grx3, a combination of 1.5 μm Grx3 and 20 μm GSH, or 9 μm Grx3.

To test whether glutathionylation of DnaK can occur under conditions mimicking oxidative stress in cells, BL21(DE3) *E. coli* cells bearing the pET28a-Smt3-DnaK plasmid were induced with 0.2 mm isopropyl 1-thio-β-d-galactopyranoside and grown at 16 °C for 16–20 h. To induce oxidative stress conditions, the cells were treated with 4 mm H_2_O_2_ at 42 °C for 15 min before harvesting and then lysing the cells in PBS buffer containing 2 mm GSSG using a JNBIO JN-3000 PLUS high-pressure cell press. The cell lysate was treated with 2 mm GSSG at 37 °C for 30 min. DnaK was then separated from the cell lysate using a nickel affinity column followed by non-reducing SDS-PAGE as described above, and cysteine modification was then detected by mass spectrometry.

MALDI-TOF and nano-LC-LTQ-Orbitrap XL MS/MS were performed to detect cysteine modifications of DnaK. Glutathionylated peptide peaks can be distinguished in MALDI-TOF spectra by the corresponding 305-Da increase in molecular mass when the samples are prepared without reducing agents such as DTT. For nano-LC-LTQ-Orbitrap XL MS/MS, trypsin-digested peptides were separated by a C_18_ reverse phase column (filled with 3-μm ReproSil-Pur C_18_-AQ from Dr. Maisch GmbH, Ammerbuch, Germany) and loaded by a C_18_ reverse phase column (filled with 5-μm ReproSil-Pur C_18_-AQ from Dr. Maisch GmbH) onto the LTQ-Orbitrap MS/MS system. Data were analyzed by Proteome Discoverer software (version 1.4.0.288, Thermo Fischer Scientific). The second MS spectra were searched in the *E. coli* database using the SEQUEST search engine. Glutathionylation or oxidation of cysteine and oxidation of methionine were set as variable modifications. The matching of searched peptide and MS spectra was filtered by Percolator calculation.

Glutathionylation or deglutathionylation of DnaK was also confirmed by the absence or presence of free thiols by staining with Alexa Fluor® 350 dye (Invitrogen) (blue fluorescence). DnaK was boiled for 10 min to destroy its secondary structure. Cooled protein was mixed with the dye and incubated in the dark at room temperature for at least 30 min. SDS-PAGE was performed to separate protein and surplus dye. Fluorescence of Alexa Fluor 350 dye was observed using excitation at 254 nm with a UV lamp.

##### Intrinsic Fluorescence

Intrinsic fluorescence measurements were carried out on a Hitachi F-4500 or a Shimadzu RF-5301PC instrument. The intrinsic fluorescence spectra of glutathionylated and deglutathionylated DnaK were measured from 300 to 400 nm using excitation wavelengths of 280 and 295 nm (to eliminate absorbance of ATP at 280 nm) at 25 °C. The proteins were prepared in Buffer B.

##### Circular Dichroism

Far-UV circular dichroism (CD) spectra were acquired between 190 and 260 nm on a Chirascan Plus CD instrument (Applied Photophysics, UK) at 25 °C in a 0.1-mm-path length thermostated quartz cuvette after preincubation for 10 min at 25 °C. Spectra of 14 μm control, glutathionylated, and deglutathionylated DnaK were measured in Buffer B.

Temperature-induced denaturation measurements were performed under the following conditions: 2.5 μm control, glutathionylated, or deglutathionylated DnaK was prepared in Buffer B in the presence or absence of 1 mm ATP. Denaturation was followed by monitoring the change in ellipticity at 222 nm. A temperature ramp of 0.5 °C/min was applied between 25 and 95 °C. All equilibrium measurements were performed using a Chirascan Plus CD instrument in a 1-mm-path length thermostated quartz cuvette. Data were collected with a band pass of 1 nm, and the sensitivity was set to 100 millidegrees. The unfolding curves were fitted to a two-state or three-state thermal unfolding model as appropriate to obtain the midpoint(s) for denaturation as described (35).

##### SEC Assay

The oligomeric states of control, glutathionylated, and deglutathionylated DnaK were compared by SEC (Superdex 200 10/300 GL column) in Buffer B at room temperature. β-Amylase (200 kDa), alcohol dehydrogenase (150 kDa), BSA (66 kDa), ovalbumin (45 kDa), carbonic anhydrase (29 kDa), PMSF-treated trypsinogen (24 kDa), and cytochrome *c* (12.4 kDa) were used as molecular mass standards. If nucleotide or NRLLLTG peptide was added, they were mixed with DnaK at room temperature 1 h before loading the samples onto the SEC column.

##### ATPase Assay (Malachite Green)

Colorimetric determination of inorganic phosphate produced by ATP hydrolysis was performed using the malachite green reagent prepared as described (36, 37). A 10-μl volume of 1 μm control, glutathionylated, or deglutathionylated DnaK was mixed with 10 μl of 2 mm ATP in Buffer B in a 96-well plate. If DnaJ, GrpE, or peptide was added, 2 μm DnaJ, 2 μm GrpE, and 200 μm NRLLLTG peptide were added individually or together to 1 μm DnaK to give a total volume of 10 μl. The plate was incubated for 4 h at 37 °C. Then 80 μl of malachite green reagent and 10 μl of 34% sodium citrate were added sequentially. The samples were mixed thoroughly and then incubated at 37 °C for 30 min before measuring the *A*_620_ on a SpectraMax M3e multimode plate reader. Intrinsic ATP hydrolysis was deducted by subtracting the signal from ATP incubated in the absence of chaperones.

##### ATP-Agarose Binding Assay

Control, glutathionylated, or deglutathionylated DnaK of 2 μm concentration in Buffer B was loaded onto an ATP-agarose column that was equilibrated with Buffer B. Non-binding protein was removed by washing with Buffer B and was collected in the run-through. Then 4 mm ADP in Buffer B was applied to elute the bound protein. The run-through and eluted fractions were checked by SDS-PAGE to evaluate the extent of binding of control, glutathionylated, or deglutathionylated DnaK with ATP/ADP.

##### Peptide Binding Assay

Peptide binding assays based on fluorescence polarization (FP) were performed as described previously (38) with slight modifications. Steady-state FP measurements were performed at room temperature after 60-min incubation in Buffer B to obtain the binding constant (*K_D_*). Binding was assessed by incubating increasing concentrations of control, glutathionylated, or deglutathionylated DnaK with a fixed concentration (20 nm) of fluorescently labeled peptide substrates (FITC-ALLLSAPRR and FITC-NRLLLTG), and FP values were measured at room temperature. FP measurements were performed in a Fluostar microreader (BMG Labtech) using the FP filter set (excitation at 485 nm and emission at 520 nm). FP values are expressed in millipolarization units. All statistical analyses were performed with Origin 8 software. Binding data were analyzed using non-linear regression analysis (single site binding model) using the equation

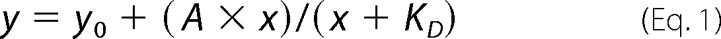
 in Origin 8. Dynamic FP measurements were performed by monitoring the FP time course to give peptide binding kinetics at room temperature. After rapid mixing of 20 nm FITC-ALLLSAPRR peptide and 1 μm control, glutathionylated, or deglutathionylated DnaK in the absence or presence of 1 mm ADP/ATP, the FP signal was recorded against time. Then 1 mm GSH with 1 mm diamide was added at the 2-h time point, and 10 mm DTT was added at the 3-h 40-min time point to glutathionylate and then deglutathionylate peptide-bound DnaK in the absence or presence of nucleotide.

##### Luciferase Refolding Assay

DnaK-assisted luciferase refolding assays were performed as described previously (39) with slight modifications. The refolding of guanidine hydrochloride-denatured firefly luciferase (Promega) was performed in Buffer B containing 1 mm ATP without DTT for 1 h at 37 °C. DnaK, DnaJ, and GrpE were added into the refolding system at final concentrations of 800, 160, and 400 nm, respectively. Each reaction was performed in triplicate, and 5 μl of the refolding mixture was removed and added to a white flat bottomed 96-well plate (JET Biofil) that was preloaded with 10 μl of Steady-Glo (Promega). After mixing, the luminescence was measured on a SpectraMax M3e multimode plate reader using a 500-ms integration time.

##### Pulldown Assay

To determine interactions between DnaJ/GrpE/σ^32^ and control/glutathionylated/deglutathionylated DnaK, pulldown assays were performed as described previously (24) with slight modifications. In brief, 2 μm His_6_-DnaJ, 5 μm His_6_-GrpE, or 2 μm His_6_-Smt3-σ^32^ and 20 μm control, glutathionylated, or deglutathionylated DnaK were incubated in Buffer B in the presence of 1 mm ADP/ATP at 8 °C for 60 min. The protein complexes were precipitated with Ni-Sepharose HP (GE Healthcare). After washing the Ni-Sepharose gel with Buffer C (50 mm Tris-HCl, pH 7.5, 300 mm KCl, 5 mm MgCl_2_) containing 40 mm imidazole, the bound proteins were eluted with Buffer C containing 300 mm imidazole. The eluted proteins were subjected to SDS-PAGE followed by staining with Coomassie Brilliant Blue to detect the interactions.

## Results

### 

#### 

##### The Single Conserved Cysteine Residue of E. coli DnaK Can Be Glutathionylated under Oxidative Stress Conditions in Vivo and in Vitro

**FIGURE 1.**
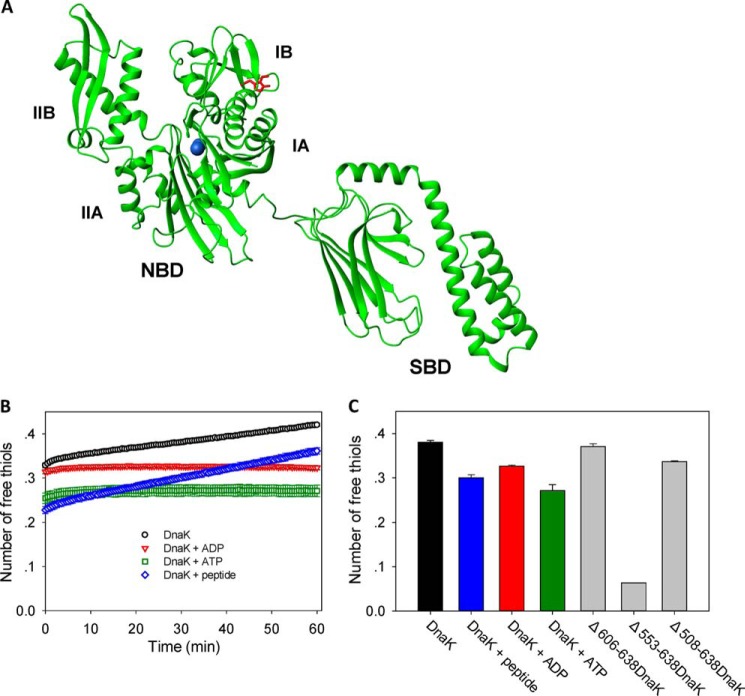
**Position and reactivity of the single cysteine residue in DnaK.** *A*, DnaK structure and locations of single cysteine (Cys-15) and tryptophan (Trp-102) residues. The NMR structure of *E. coli* DnaK (Protein Data Bank code 2kho) is displayed in *green*, the Cys residue is in *blue*, and the Trp residue is in *red*. The N-terminal NBD and its subdomains IA, IB, IIA, and IIB and the C-terminal SBD are indicated. *B*, the time course of cysteine reactivity of DnaK in the absence and presence of ADP, ATP, or peptide was monitored in a Fluostar plate reader. The number of free thiols per DnaK molecule was measured by DTNB assay as an indicator of cysteine reactivity. The number of free thiols was calculated from the absorbance at 412 nm using a standard curve. The data shown are the mean of three independent measurements, and the *error bars* represent the S.E. *C*, the number of free thiols measured at the 30-min time point is plotted to allow comparison of cysteine reactivity of WT DnaK in the absence and presence of ADP, ATP, or peptide and with the truncation mutants Δ606–638DnaK, Δ553–638DnaK, and Δ508–638DnaK. The data shown are the mean of three independent measurements, and the *error bars* represent the S.E.

**FIGURE 2.**
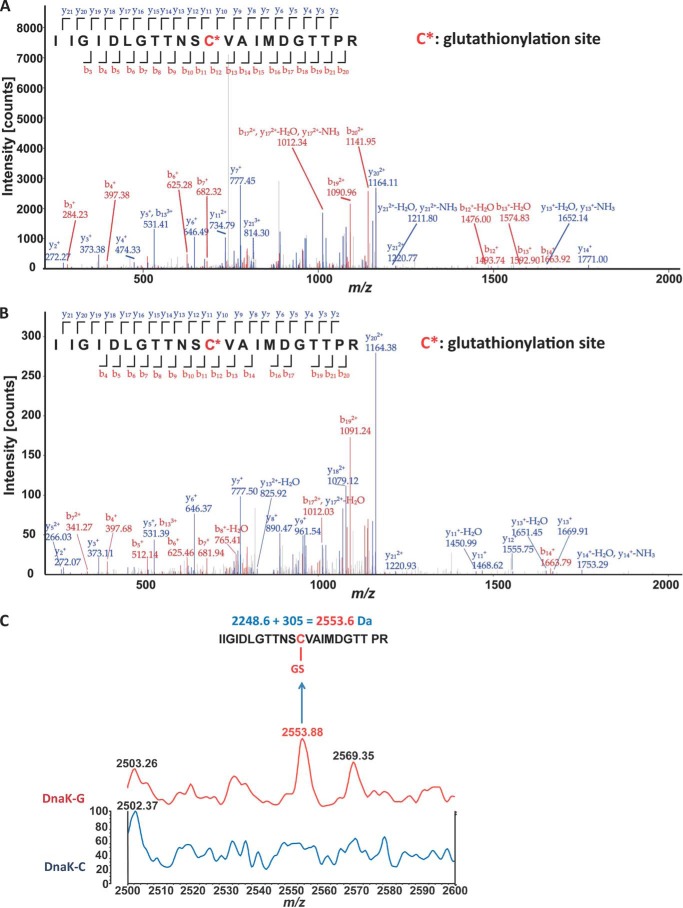
**Detection of glutathionylation at Cys-15 of DnaK by mass spectrometry.** *A* and *B*, nano-LC-LTQ-Orbitrap XL analysis of 2553-Da glutathionylated peptide after trypsin digestion. The detected peaks (*main panel*) correspond to the predicted peptides (*inset*) where *red* corresponds to observed N-terminal peptide fragments and *blue* corresponds to observed C-terminal peptide fragments. C* indicates Cys-15, which undergoes glutathionylation. *A*, DnaK from GSSG-treated lysates of *E. coli* cells that had been subjected to oxidative stress; *B*, DnaK treated with diamide and GSH after purification. A full list of observed thiol modifications is shown in Table 2. For further details, see “Experimental Procedures.” *C*, MALDI-TOF detection of glutathionylation of DnaK treated with diamide and GSH after purification. Mass spectra for untreated control (*DnaK-C*) and glutathionylated (*DnaK-G*) samples are shown.

*E. coli* DnaK has only one cysteine residue, Cys-15, which lies on the β-sheet surface of the IA subdomain of the ATPase domain (Fig. 1*A*). The cysteine at this position is highly conserved among Hsp70 family members, suggesting that it may be important for function. There is evidence from the literature that the Cys residue of DnaK is reactive and can be modified under oxidative stress conditions *in vivo*. For example, sulfinic, sulfenic, and sulfonic modifications of DnaK were observed in *E. coli* cells treated with H_2_O_2_ (27), and *S*-nitrosylation of DnaK was detected in *S*-nitrosoglutathione-treated cell extracts (28). Furthermore, *S. enterica* Typhimurium DnaK, which has 97% homology with *E. coli* DnaK, was found to be glutathionylated and cysteinylated *in vivo* (40). To confirm that glutathionylation of *E. coli* DnaK can occur under conditions mimicking oxidative stress *in vivo*, we treated *E. coli* cells with H_2_O_2_ and then lysed the cells in the presence of GSSG before extracting the DnaK protein for analysis by mass spectrometry. Glutathionylation and sulfonic modifications could be detected for DnaK from *E. coli* cell lysates (Fig. 2*A* and Table 2).

**TABLE 2:**
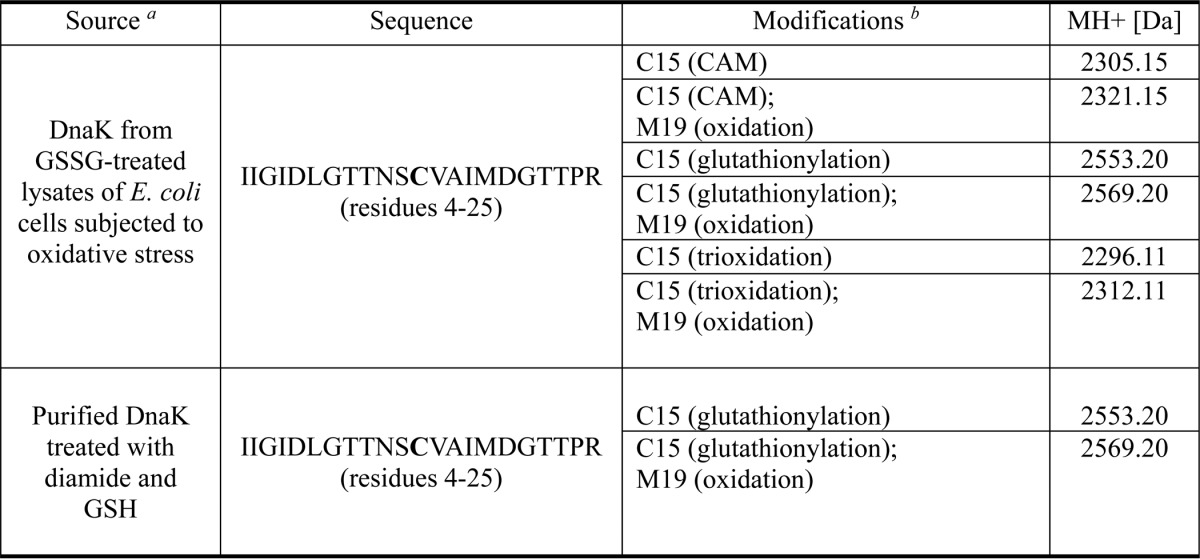
**Nano-LC-LTQ-Orbitrap XL MS/MS detection of thiol modifications of DnaK**

*^a^* For further details see “Experimental Procedures.”

*^b^* Free cysteine residues in the DnaK sample were blocked with carbamidomethyl (CAM) before tryptic digestion and mass spectrometry analysis.

To further understand the susceptibility of DnaK to undergo glutathionylation, the cysteine reactivity of *E. coli* DnaK was investigated. The p*K_a_* value of the cysteine residue in DnaK was obtained experimentally by detecting formation of the thiolate anion by monitoring absorbance over a range of pH values and was found to be about 12. This agrees well with the predicted value of 12.04 obtained using Propka software. We also modeled the structure of *S. enterica* Typhimurium DnaK and found that the predicted p*K_a_* value of its single cysteine residue is 12.7. The high p*K_a_* value indicates that although both *S. enterica* Typhimurium (40) and *E. coli* DnaK (27) homologues are reported to undergo cysteine modifications *in vivo* it is likely that the cysteine residue of DnaK will only become oxidized under relatively extreme (*i.e.* oxidative stress) conditions *in vivo*.

The DTNB assay is often used to detect exposed thiol groups (32). At room temperature, the cysteine reactivity of apo-DnaK measured by DTNB assay was about 0.3 initially but rose with time (Fig. 1*B*). The presence of ADP, ATP, or peptide inhibited cysteine reactivity of DnaK to different degrees (Fig. 1, *B* and *C*). Cysteine reactivity of nucleotide-bound DnaK remained steady over time, whereas cysteine reactivity of peptide-bound DnaK rose with time like apo-DnaK (Fig. 1*B*). Truncation of residues 553–638 of DnaK (Δ553–638DnaK) destroys the SBDα lid structure, causing it to bind to the SBDβ, and thus this mutant mimics substrate-bound DnaK (5, 41). We found that Δ553–638DnaK had very low cysteine reactivity (Fig. 1*C*). In contrast, Δ606–638DnaK, which maintains the intact SBDα lid structure, and Δ508–638DnaK, which lacks the SBDα lid (5, 42), both had comparable cysteine reactivity to WT DnaK (Fig. 1*C*). These results are consistent with an inhibitory effect of substrate binding on cysteine reactivity of DnaK. The increasing cysteine reactivity over time is consistent with the dynamic nature of the nucleotide-depleted NBD of DnaK, whereas constant cysteine reactivity over time corresponds to the more stable structure of the nucleotide-bound NBD of DnaK (43, 44). Cysteine reactivity within the NBD decreased upon substrate binding to the SBD, which reflects domain communication between the NBD and SBD of DnaK. Therefore nucleotide binding to the NBD or substrate binding to the SBD may cause burial of the NBD cysteine residue and protection of the cysteine from being modified, which suggest that cysteine modification of *E. coli* DnaK will only occur under relatively extreme physiological conditions, consistent with the conclusion of p*K_a_* measurements above.

**FIGURE 3.**
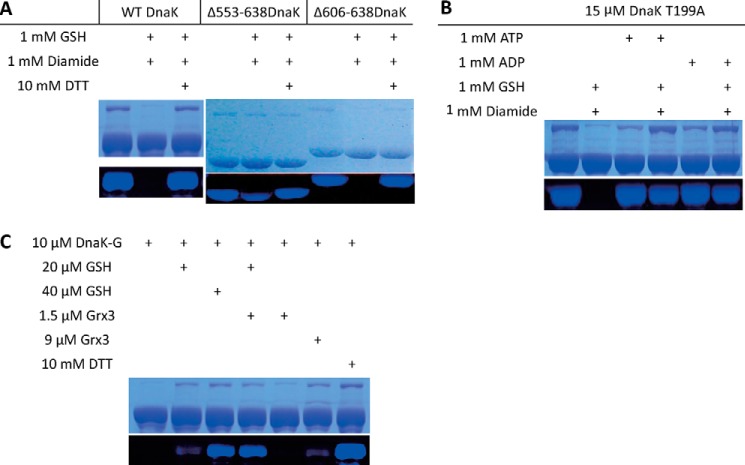
**Factors affecting the extent of glutathionylation of DnaK.** Alexa Fluor 350 dye staining was used to detect the presence or absence of a free thiol in DnaK or its mutants in non-glutathionylated and glutathionylated states. Unmodified cysteine residues for untreated control, glutathionylated, and deglutathionylated samples were labeled with fluorescent dye. Equal loading of samples is shown by Coomassie Brilliant Blue staining of the same SDS-PAGE gel. *A*, WT DnaK, Δ553–638DnaK, and Δ606–638DnaK before and after treatment with diamide and GSH. The C-terminal truncation mutant Δ553–638DnaK mimics substrate binding. *B*, DnaK in the absence or presence of ATP or ADP before and after treatment with diamide and GSH. The DnaK mutant T199A binds but does not hydrolyze ATP. *C*, catalytic deglutathionylation of DnaK by Grx3 in the presence of GSH. Glutathionylated DnaK (*DnaK-G*) was treated with a range of concentrations of reducing agents and Grx3 enzyme alone or in combination as indicated.

To characterize the effects of glutathionylation on DnaK structure and function, it was necessary to first obtain homogeneously glutathionylated DnaK. We therefore tested the efficiency of a range of glutathionylation conditions *in vitro*. We also tested various methods to determine the presence and extent of glutathionylation. Although glutathionylation of proteins can often be confirmed by Western blotting, we consistently found that glutathionylation of DnaK cannot be recognized readily by anti-GSH antibody. However, as used above to confirm the presence of glutathionylated DnaK in *E. coli* cell extracts (Fig. 2*A*), the presence of glutathionylated peptide was confirmed by nano-LC-LTQ-Orbitrap XL MS/MS (Fig. 2*B*), and MALDI-TOF MS detection of trypsin-digested modified peptide also confirmed the expected 305-Da increase in molecular mass upon glutathionylation (Fig. 2*C*). To provide a sensitive means to measure the extent of glutathionylation of DnaK, we used the maleimide functionalized dye Alexa Fluor 350, which reacts specifically with reduced cysteine residues. The inability of glutathionylated DnaK to bind Alexa Fluor 350 dye confirmed that the modification of cysteine was complete, whereas control DnaK and deglutathionylated DnaK reacted with the dye highly efficiently (Fig. 3*A*).

We tested a wide range of glutathionylation conditions using typical glutathionylation reagents such as GSSG, GSH with H_2_O_2_, and GSH with diamide. Diamide is highly efficient in promoting disulfide bond formation (45). If there is sufficient GSH, exposed thiol residues in a protein rapidly become glutathionylated in the presence of diamide (45). Consistent with the results in cell extracts treated with GSSG above (Fig. 2*A*), all these reagents led to glutathionylation of DnaK, but the reaction was most rapid with GSH and diamide (data not shown), and so the reaction conditions with diamide and GSH were optimized further. We found that a 4–6-h reaction at room temperature produced only partial glutathionylation of untagged DnaK as indicated by Alexa Fluor 350 dye staining (data not shown), whereas a 1-h reaction at the higher temperature of 37 °C gave complete glutathionylation (Fig. 3*A*). In the presence of ADP or ATP, no glutathionylation of DnaK occurred (Fig. 3*B*; to avoid hydrolysis of ATP catalyzed by WT DnaK, DnaK T199A (46) was used instead of WT DnaK for this experiment). However, in the presence of peptide, DnaK could still be partially glutathionylated (data not shown), and likewise, the C-terminal truncation mutant Δ553–638DnaK, which mimics the substrate-bound state, can also be partially glutathionylated, whereas the C-terminal truncation mutant Δ606–638DnaK, which only lacks the C-terminal random coil region, can be completely glutathionylated like WT DnaK (Fig. 3*A*). These results suggest that the dynamics of the DnaK NBD significantly influences the degree of exposure of Cys-15 and its susceptibility to modification.

We also tested reversal of the glutathionylation reaction by reducing agents with or without addition of the glutaredoxin enzyme Grx3. The results show that a combination of Grx3 and GSH worked synergistically to cause deglutathionylation of DnaK (Fig. 3*C*), indicating the possibility that Grx-dependent deglutathionylation of DnaK may occur *in vivo*.

##### Glutathionylation Induces Reversible Structural and Conformational Changes in DnaK

**FIGURE 4.**
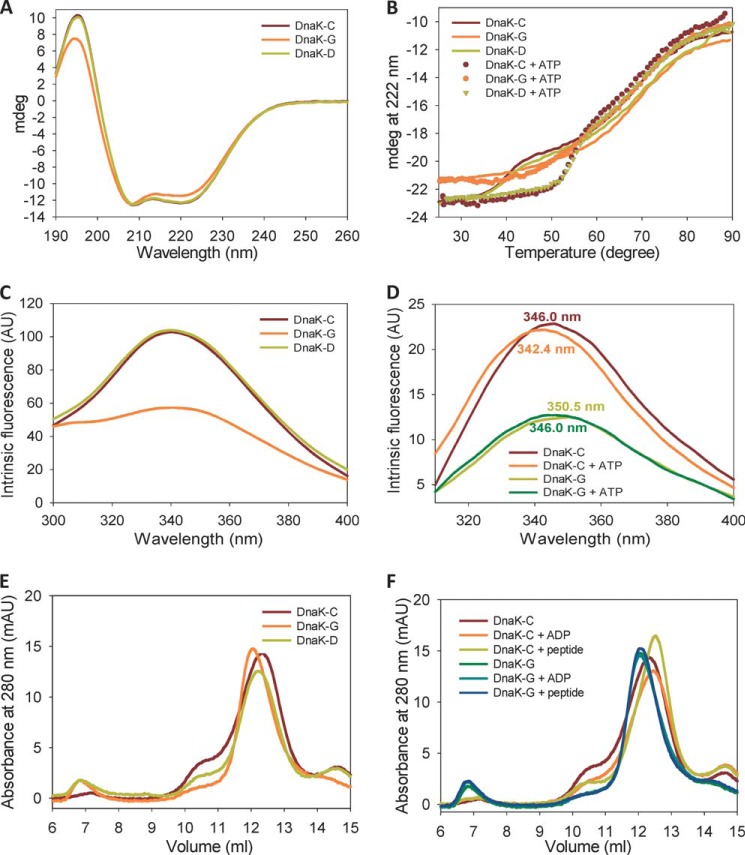
**Effect of glutathionylation on structure and allosteric conformational changes of DnaK.** Data are shown for untreated control (*DnaK-C*), glutathionylated (*DnaK-G*), and deglutathionylated (*DnaK-D*) samples of DnaK with or without peptide or nucleotides as indicated. Samples were in Buffer B. *AU*, arbitrary units; *mAU*, arbitrary units/1000; *mdeg*, millidegrees. *A*, secondary structure differences were monitored by acquiring far-UV CD spectra for 14 μm DnaK in a 0.1-mm cuvette. *B*, thermal denaturation was monitored by the CD signal at 222 nm for 2.5 μm DnaK in a 1-mm cuvette. *C*, intrinsic fluorescence spectra of 2 μm DnaK were measured using an excitation wavelength of 280 nm. *D*, conformational change of DnaK induced by ATP was monitored by comparing differences in the intrinsic fluorescence spectra of 2 μm DnaK in the absence and presence of ATP using an excitation wavelength of 295 nm. *E*, the oligomeric state of DnaK was determined by SEC. *F*, conformational change of DnaK induced by nucleotide or peptide was monitored by comparing differences in the elution peak using SEC.

The CD spectrum of DnaK shows two obvious peaks at 222 and 208 nm, reflecting the high α-helical content of the DnaK structure. Glutathionylation of DnaK causes a decrease in the CD signal at 222 nm, whereas the spectrum of deglutathionylated DnaK is identical to that of the untreated control (Fig. 4*A*). This indicates that glutathionylation causes partial disruption of the α-helical structure in DnaK, and this structural change is reversed upon deglutathionylation. The α-helix that lies opposite the β-sheet surface containing Cys-15 is most likely to be affected (Fig. 1*A*). This α-helix is very important for domain communication (47).

Thermal denaturation monitored by the CD signal at 222 nm was also used to evaluate the stability of DnaK. ATP-bound DnaK showed higher stability against heat denaturation than apo-DnaK (Fig. 4*B* and Table 3), which agrees with previously reported results (44, 48). Unmodified DnaK shows a three-state thermal denaturation curve (Fig. 4*B*). The first transition is attributed to the unfolding of the NBD, and the second transition represents the unfolding of the SBD (44). We found that for glutathionylated DnaK in the apo state the first transition disappeared, whereas the only observed transition has a midpoint similar to the second transition of untreated control DnaK (Fig. 4*B* and Table 3). In contrast, when ATP was present, glutathionylated DnaK again showed a three-state transition with similar midpoint values as untreated control DnaK (Table 3), although the unfolding curves do not entirely overlay (Fig. 4*B*). This result suggests that glutathionylation perturbs the structure of the NBD, whereas binding of ATP to glutathionylated DnaK restores the structure of the NBD to some extent, which is consistent with previous results (27). The stability of the SBD was not obviously affected by glutathionylation or the presence of ATP as is indicated by similar midpoint values for the second transition (Table 3). The thermal denaturation curves and calculated midpoint values for deglutathionylated DnaK and the untreated control were almost identical whether in the absence or presence of ATP (Fig. 4*B* and Table 3).

**TABLE 3:**
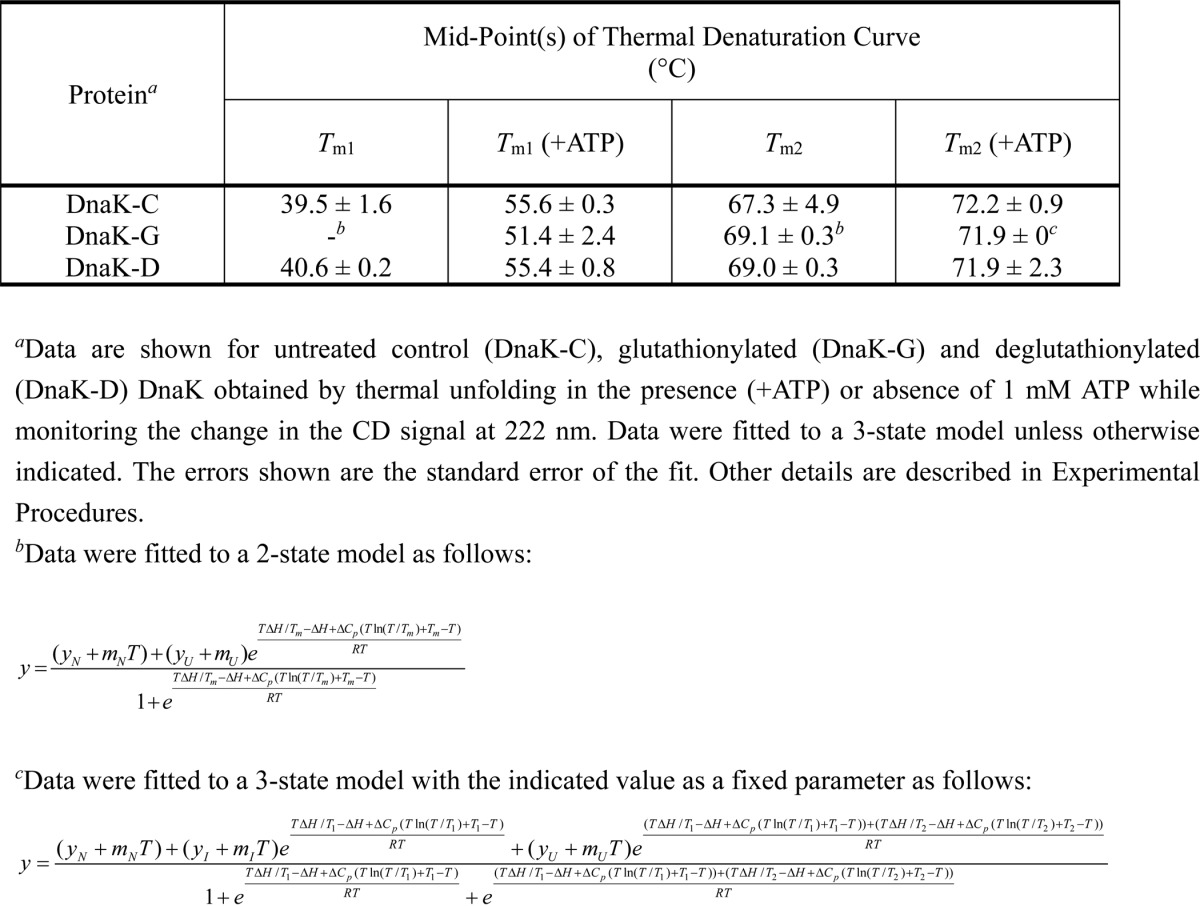
**Effect of glutathionylation on midpoint values for thermal denaturation of DnaK in the presence and absence of nucleotide**

DnaK has only one tryptophan residue, which is also located in the NBD (Fig. 1*A*). The intrinsic fluorescence of this tryptophan can be used to monitor structural changes in the NBD. We found that glutathionylation of DnaK caused a significant decrease in intrinsic fluorescence intensity, which is reversed on deglutathionylation (Fig. 4*C*), suggesting that glutathionylation causes a change in the tertiary conformation of DnaK such as unfolding of the NBD. Addition of ATP caused a slight blue shift of the intrinsic fluorescence peak for both control DnaK (from 346.0 to 342.4 nm; Fig. 4*D*) and glutathionylated DnaK (from 350.5 to 346 nm; Fig. 4*D*). The peak shift of glutathionylated DnaK upon ATP binding may reflect partial restoration of the NBD structure as judged by the similar peak position of ATP-bound glutathionylated DnaK and apo control DnaK, although the restoration of fluorescence intensity was less apparent.

DnaK tends to show different oligomeric states in the absence and presence of ADP, ATP, or peptide (49) with the presence of nucleotide and/or peptide favoring the monomeric state of DnaK (Fig. 4*F*). In SEC analysis, glutathionylation of DnaK causes a reduction in the elution volume (Fig. 4*E*), which indicates a higher oligomeric state and/or a loosening of the structure of DnaK. In the presence of ADP or peptide, the elution peak of glutathionylated DnaK did not shift (Fig. 4*F*). We also tested the effect of ATP on the SEC elution profile of glutathionylated DnaK using the DnaK mutant T199A, which binds ATP but has extremely weak ATPase activity. Similarly, no shift of the elution peak was observed in the presence of ATP (data not shown).

All the above results demonstrate that the structural and conformation changes in DnaK induced by glutathionylation are completely reversible. Conversely, although the intrinsic fluorescence and thermal unfolding monitored by CD indicated that ATP could partially rescue the perturbed NBD structure of glutathionylated DnaK, the results monitored by SEC fail to detect an obvious effect of the presence of nucleotides or substrate peptide on the conformation of glutathionylated DnaK in contrast to the effects seen for unmodified DnaK. One possible reason is that glutathionylated DnaK has a weaker ability to bind nucleotides or peptide. The alternative explanation is that the allosteric conformational changes in DnaK are reduced upon glutathionylation. These possibilities are addressed below.

##### Glutathionylation Reversibly Regulates the ATPase Activity of E. coli DnaK

**FIGURE 5.**
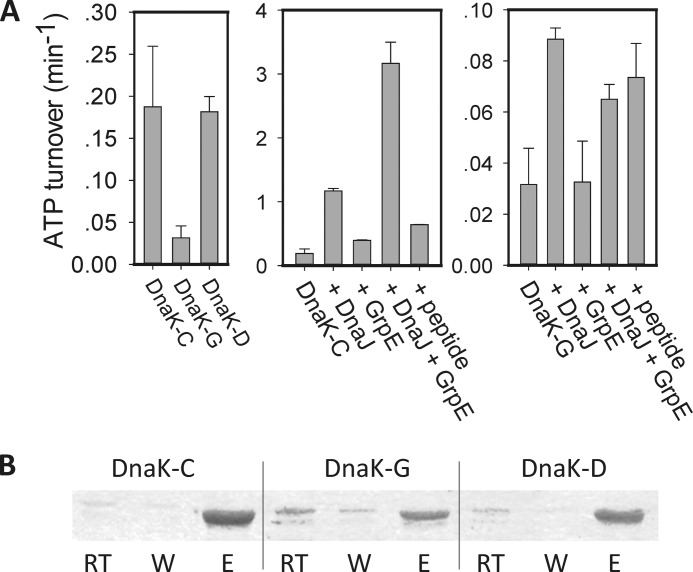
**Effect of glutathionylation on ATPase activity and nucleotide binding of DnaK.** Data are shown for untreated control (*DnaK-C*), glutathionylated (*DnaK-G*), and deglutathionylated (*DnaK-D*) samples of DnaK with or without the co-chaperone DnaJ, nucleotide exchange factor GrpE, or peptide as indicated. Details of reaction conditions are described under “Experimental Procedures.” *A*, effect of glutathionylation on ATPase activity of DnaK measured by malachite green assay. The data shown are the mean of three independent experiments, and the *error bars* represent the S.E. *B*, effect of glutathionylation on ATP and ADP binding to DnaK. Binding to ATP-agarose and elution by ADP were detected by SDS-PAGE. *RT*, run-through; *W*, wash; *E*, eluate.

ATPase activity of DnaK is important for regulating substrate binding and release and is essential for the foldase activity of DnaK (39). DnaJ, GrpE, and peptide can greatly promote the ATPase activity of DnaK (10, 36). We found that glutathionylated DnaK only possessed 15–20% ATPase activity compared with deglutathionylated DnaK or the untreated control (Fig. 5*A*). This suggests that ATP binding to DnaK is weaker or the ATPase site is partially disrupted upon glutathionylation within the NBD. DnaJ can accelerate ATP turnover of glutathionylated DnaK 2-fold compared with a 6-fold increase for unmodified DnaK at the same DnaJ to DnaK ratio (2:1) (Fig. 5*A*). GrpE cannot promote ATPase activity of glutathionylated DnaK, and the combination of GrpE and DnaJ had a similar effect on the ATPase activity of glutathionylated DnaK as DnaJ alone (Fig. 5*A*). At the same high peptide to DnaK ratio (200:1), peptide promoted the ATPase activity of glutathionylated DnaK to a similar degree as control DnaK (Fig. 5*A*), which indicates that domain communication and allostery upon peptide binding are retained in glutathionylated DnaK. We can therefore conclude that glutathionylated DnaK is still able to bind DnaJ and peptide, but the cooperative effects of DnaJ and GrpE on DnaK are reduced upon glutathionylation.

To check whether glutathionylation also weakens ATP binding to DnaK, we performed an ATP-agarose binding assay. Analyzing the amount of non-bound protein present in the run-through, we found that the amount of DnaK that binds to ATP-agarose is reduced for glutathionylated DnaK compared with deglutathionylated DnaK or the unmodified control protein (Fig. 5*B*). Analyzing the bound protein eluted by ADP, we found that there was still glutathionylated DnaK present in the eluted fraction (Fig. 5*B*). Therefore glutathionylated DnaK can still bind nucleotide, but the binding is weaker than in deglutathionylated DnaK or the unmodified control. Conformational and secondary structural changes induced by glutathionylation may contribute to both weaker ATP binding ability and lower catalytic efficiency at the ATPase site.

##### Glutathionylation Reversibly Regulates the Peptide Binding and Foldase Activity of DnaK

**FIGURE 6.**
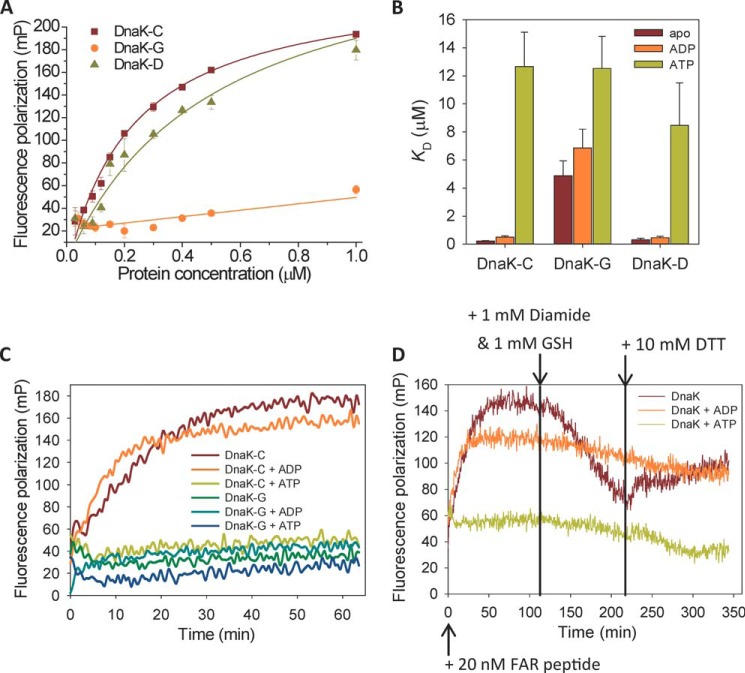
**Effect of glutathionylation on peptide binding to DnaK.** Data are shown for untreated control (*DnaK-C*), glutathionylated (*DnaK-G*), and deglutathionylated (*DnaK-D*) samples of DnaK with or without nucleotides as indicated. Experiments were performed in Buffer B. *A*, fluorescence polarization at 520 nm after excitation at 485 nm was used to monitor the binding of 20 nm FITC-labeled ALLLSAPRR peptide to different concentrations of DnaK in the absence of nucleotide with or without glutathionylation as indicated. The data shown are the mean of three independent experiments, and the *error bars* represent the S.E. *B*, the affinity constants (*K_D_*) for binding of DnaK to ALLLSAPRR peptide in apo, ADP, and ATP states as indicated were calculated by fitting the binding curve (see “Experimental Procedures”) measured by fluorescence polarization for different concentrations of DnaK with or without glutathionylation as indicated. The data points in each binding curve were the mean of three independent experiments, and the *error bars* shown represent the S.E. of the fit. *C*, kinetics of 20 nm peptide binding to 1 μm DnaK before or after glutathionylation in apo, ADP, or ATP states as indicated. *D*, effect on kinetics of peptide binding to DnaK of glutathionylation (1 mm GSH and 1 mm diamide) and deglutathionylation (10 mm DTT) conditions introduced at the time points indicated. Fluorescence polarization at 520 nm after excitation at 485 nm was used to monitor the binding of 20 nm ALLLSAPRR peptide to 1 μm DnaK in apo, ADP, and ATP states as indicated. *FAR*, FITC-ALLLSAPRR peptide; *mP*, millipolarization units.

Synthetic peptides have been used to map high affinity binding motifs in substrates of Hsp70, leading to the identification of a number of high affinity peptides such as NRLLLTG and ALLLSAPRR, and peptide binding ability is often used to represent substrate binding capability (50, 51). FP can be used to measure binding of small fluorescently labeled peptides to Hsp70 (38, 52). In this study, FITC-ALLLSAPRR and FITC-NRLLLTG peptides were tested and gave similar results (FITC-ALLLSAPRR data are shown in Fig. 6; FITC-NRLLLTG data not shown). We found that glutathionylation of DnaK caused an obvious decrease in binding affinity with peptide substrate (Fig. 6, *A* and *B*). Before glutathionylation and after deglutathionylation, the binding constant of short peptides for binding to DnaK was around 0.2–0.5 μm, which is comparable with previous reports (38), whereas for glutathionylated DnaK it was 4–6 μm (Fig. 6*B*). The effect of glutathionylation within the NBD of DnaK resulting in a reduction in affinity for peptide binding suggests that glutathionylation induces changes in local structure that are communicated allosterically to the SBD. ADP binding to DnaK did not obviously alter the binding affinity of peptide (Fig. 6*B*), which is consistent with the finding that ADP does not alter the undocking of the NBD and SBD compared with apo-DnaK (2, 5). ATP binding to DnaK decreased the affinity for peptide to give a binding constant of at least 10 μm (Fig. 6*B*), which is consistent with previous reports of docking of the NBD and SBD in the presence of ATP (3–5). In summary, we found that peptide binding to glutathionylated DnaK is similarly weak in the apo and ADP states. Upon ATP binding to the NBD, the affinity of peptide binding for glutathionylated DnaK is similar to the affinity for untreated control DnaK. This suggests that glutathionylated DnaK retains the response to ATP binding in terms of docking of the NBD and SBD. However, normal undocking of the NBD and SBD in the apo or ADP-bound states is impaired for glutathionylated DnaK.

When FP was applied to monitor the time course of peptide binding in the presence or absence of ADP/ATP, it was observed that binding of peptide to DnaK required at least 30 min to reach a maximum in the absence or presence of ADP, whereas it reached a maximum too rapidly to be measured by plate reader in the presence of ATP (Fig. 6*C*). However, whether in the apo state or in the ADP/ATP state, glutathionylated DnaK showed a similar time course as the ATP-bound state of unmodified DnaK (Fig. 6*C*). The rapid peptide binding of ATP-bound DnaK is due to the tendency of the SBDα lid to be in the open position. This indicates that glutathionylation may also affect opening and closing of the SBDα lid. When peptide binding to unmodified apo- or nucleotide-bound DnaK reached a maximum, diamide and GSH were added to test whether glutathionylation would then weaken the peptide binding and decrease the FP value. It was observed that, only when DnaK is in the nucleotide-free state, the peptide binding curve shows a decreased FP value upon addition of cysteine modifier and then shows an increase in FP value upon subsequent addition of DTT (Fig. 6*D*). The eventual slight decrease in FP value in the peptide binding curve for the ADP/ATP-bound DnaK is due to the slight attenuation of fluorescence over long time periods. It was confirmed that DnaK can still be glutathionylated in the presence of peptide but not in the presence of ADP or ATP, supporting previous reports (27) and our results obtained using Alexa Fluor 350 dye staining (Fig. 3*B*).

**FIGURE 7.**
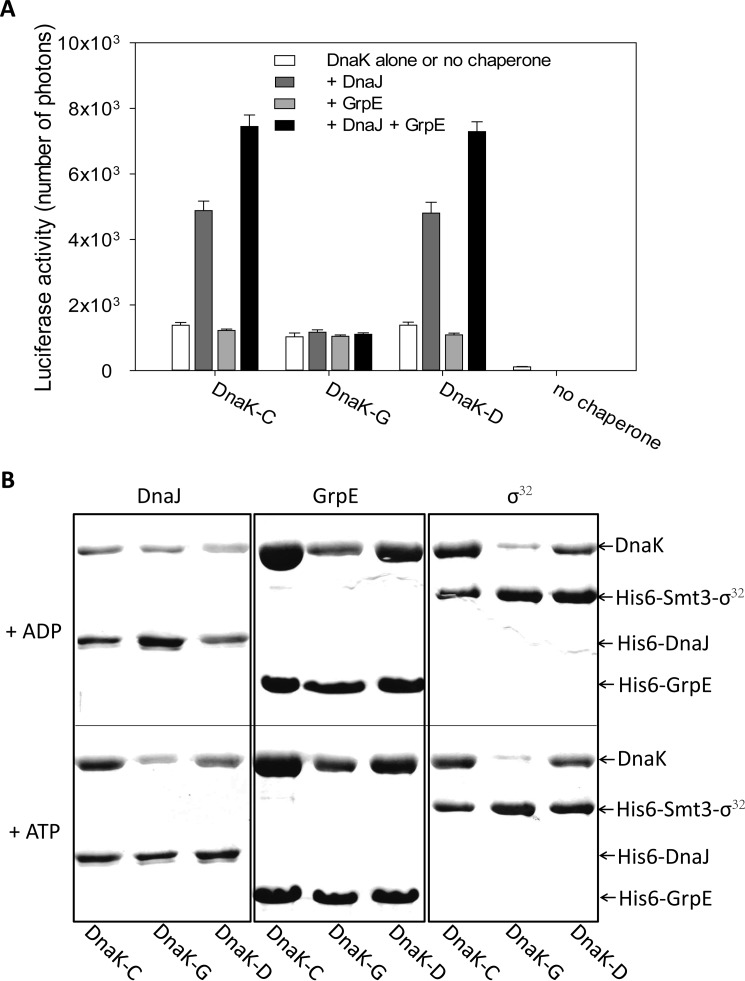
**Effect of glutathionylation on the function of DnaK.** *A*, effect of glutathionylation on luciferase refolding activity of DnaK. Refolding of chemically denatured firefly luciferase by untreated control (*DnaK-C*), glutathionylated (*DnaK-G*), and deglutathionylated (*DnaK-D*) samples of DnaK in the absence and presence of DnaJ and/or GrpE was measured using the Steady-Glo luciferase assay system (Promega); 80 nm luciferase, 800 nm DnaK, 160 nm DnaJ, and 400 nm GrpE (in Buffer B) were added into the refolding system. Luciferase activity is plotted as the number of photons produced. The data shown are the mean of three independent experiments, and the *error bars* represent the S.E. *B*, effect of glutathionylation on interaction of DnaK with DnaJ, GrpE, and σ^32^ detected by pulldown assay. Samples of 20 μm untreated control (*DnaK-C*), glutathionylated (*DnaK-G*), or deglutathionylated (*DnaK-D*) DnaK were incubated with 2 μm His_6_-DnaJ, 5 μm His_6_-GrpE, or 2 μm His_6_-Smt3-σ^32^ at 8 °C for 60 min in the presence of 1 mm ADP or ATP as indicated. Elution with Buffer C containing 300 mm imidazole from Ni-Sepharose HP was detected by SDS-PAGE and staining with Coomassie Brilliant Blue.

The finding that peptide binding to DnaK is impaired by glutathionylation suggests that the substrate binding ability of DnaK is reduced by glutathionylation. To further investigate this, the effect of glutathionylation on the foldase activity of DnaK was examined using the luciferase refolding assay. The intrinsic foldase activity of DnaK is relatively weak and was facilitated by DnaJ or a combination of DnaJ and GrpE but not by GrpE alone (Fig. 7*A*), consistent with previous reports (39). We found that the intrinsic foldase activity of glutathionylated DnaK was comparable with control DnaK and deglutathionylated DnaK, but DnaJ or a combination of DnaJ and GrpE could not promote it (Fig. 7*A*). The decoupling of the effects of DnaJ/GrpE on luciferase refolding confirmed that interaction of DnaK with DnaJ or GrpE was impaired by glutathionylation as the ATPase assay result also suggested (Fig. 5*A*). Reduced ATPase activity and reduced flexibility of the structure of glutathionylated DnaK may contribute to the reduction in foldase activity and loss of cooperation between DnaK and its co-chaperones.

##### Glutathionylation Reversibly Regulates the Interaction of DnaK with DnaJ, GrpE, and σ^32^

The results of the ATPase and luciferase refolding assays suggest that glutathionylation of DnaK impairs its interaction with DnaJ and GrpE. To confirm this, we performed a pulldown assay to directly analyze interaction of glutathionylated DnaK with DnaJ or GrpE. DnaK can interact with DnaJ in the presence of ADP or ATP, but DnaJ binds more strongly to ATP-bound DnaK (Fig. 7*B*). We found that in the presence of ADP interaction of DnaK with DnaJ was not obviously affected by glutathionylation, but in the presence of ATP, DnaJ bound less well to glutathionylated DnaK than control DnaK or deglutathionylated DnaK (Fig. 7*B*). The binding affinity of GrpE for DnaK is very high, and the reported *K_D_* is less than 30 nm (11, 53). GrpE shows similar binding ability to ADP-bound and ATP-bound DnaK (Fig. 7*B*). We found that GrpE binds less well to glutathionylated DnaK than control DnaK or deglutathionylated DnaK in the presence of either ADP or ATP, although the effect of glutathionylation on interaction of GrpE and DnaK was slightly stronger in the presence of ADP than that in the presence of ATP (Fig. 7*B*). The decreased binding ability of DnaJ in the presence of ATP and GrpE in the presence of ADP to glutathionylated DnaK may contribute to weaker promotion of ATPase activity and foldase activity by DnaJ and/or GrpE.

To address the significance of the loss of substrate binding ability caused by glutathionylation of DnaK, we chose to look at σ^32^ as a physiological binding partner to investigate a possible link between oxidative stress and the heat shock response. We found that σ^32^ had lower binding affinity for glutathionylated DnaK compared with control DnaK or deglutathionylated DnaK in the presence of ADP or ATP (Fig. 7*B*). (It has been reported that σ^32^ binds more strongly to ADP-bound DnaK than to ATP-bound DnaK (24), although in this study the binding affinity of σ^32^ for ADP-bound and ATP-bound DnaK appeared to be similar (Fig. 7*B*).) Our results suggest that glutathionylation of DnaK may help activation of σ^32^ by causing its release.

## Discussion

The effect of post-translational modifications on protein function is increasingly recognized as an important physiological regulatory mechanism. The glutathionylation/deglutathionylation cycle of cysteine residues depends on the redox state of the microenvironment, and reversible glutathionylation may help protect proteins from irreversible thiol oxidation reactions as well as providing a mechanism to transfer redox information (54). The observation of cysteine modifications of DnaK *in vivo* (27, 28, 40, 55) and the loss of DnaK function under oxidative stress conditions (27) suggest that modifications such as glutathionylation may play a role in modulating the function of DnaK. This study aimed to elucidate the effect of glutathionylation on the structure and function of DnaK and revealed that decreased chaperone activity of glutathionylated DnaK can be attributed to perturbation of its structure and reduction in nucleotide and substrate binding ability as well as impaired cooperation with co-chaperones. How DnaK function is perturbed by glutathionylation under oxidative conditions and fully restored by deglutathionylation under reducing conditions is described in this study with the results suggesting that thiol modification may be an important regulation mechanism for DnaK. This study also explored the interaction of glutathionylated DnaK with one of its client proteins, σ^32^, implying a physiological role of DnaK glutathionylation. We observed impaired binding of σ^32^ to glutathionylated DnaK, indicating that DnaK may act as a redox sensor via cysteine modification to transfer redox information and regulate the heat shock response.

It is generally acknowledged that Hsp70 mainly exists in the nucleotide-bound state *in vivo* due to its high affinity for ATP/ADP and the abundance of nucleotide in the cell. Nucleotide binding protects Hsp70 from undergoing thiol modification within the NBD, which is not only verified for DnaK in this study and a previous report (27) but also for yeast Hsp70 Ssa1 (56) and human inducible Hsp72,^4^ suggesting that nucleotide binding protects the Hsp70 NBD from thiol attack and helps maintain its normal chaperone activity. ATP depletion under oxidative stress in different organisms was first observed nearly 30 years ago (27, 57–59). Thus oxidative stress may dramatically increase the frequency of thiol modification of Hsp70, especially within the NBD. Thiol modification of DnaK in *E. coli* (27), *S. enterica* serovar Typhimurium (40), and Firmicutes bacteria (55) confirms the existence of the apo state of DnaK in bacteria.

This study found that the normal growth temperature of *E. coli* (37 °C) was sufficient for complete glutathionylation of apo-DnaK to occur, and heat stress temperatures (over 40 °C) are not essential. Therefore the requirement for heat stress temperatures for cysteine modification of DnaK *in vivo* (27) may be primarily related to accelerated nucleotide depletion rather than a prerequisite for partial unfolding of DnaK as previously thought. The regulatory effect of glutathionylation on DnaK suggests that cysteine modification may be an important mechanism for regulation of Hsp70 function. Cysteine modification is not only related to delivery of redox information but is also involved in the normal activity of some proteins. Almost all known Hsp70 family members have at least one cysteine residue, and the number of cysteine residues is observed to increase moving from bacteria to yeast and then mammals, which suggests that cysteine residues in Hsp70 may be involved in adaptation to increasingly complex or specialized cellular environments. The yeast Hsp70 member Ssa1 was found to be modifiable by *N*-ethylmaleimide in the absence of nucleotide, and this modification reduces transmembrane transport as well as the ATPase and luciferase refolding activities of Ssa1 (56, 60, 61). 4-Hydroxy-2-nonenal modification at Cys-267 of mammalian Hsp72 inhibits its protein refoldase activity (62). The ATPase activity of Hsp72 is inhibited by oxidative modification of Cys-306, and modification of this cysteine distinguishes redox sensing of inducible Hsp72 and constitutive Hsp73 (63). The human endoplasmic reticulum-resident Hsp70 family member BiP can form an intramolecular disulfide bond, which enhances its substrate binding ability in an oxidative environment (64), and the cysteine in yeast BiP homologue Kar2 can also be oxidized, promoting its chaperone activity (65). Glutathionylation and *S*-nitrosylation of different Hsp70 homologues have been detected by proteomics methods (66–71). Glutathionylation of Hsc70 was found to enhance inhibition of heat-induced luciferase aggregation (68). These findings point to cysteine modification, including glutathionylation, as playing an essential role in some specific functions of Hsp70.

**FIGURE 8.**
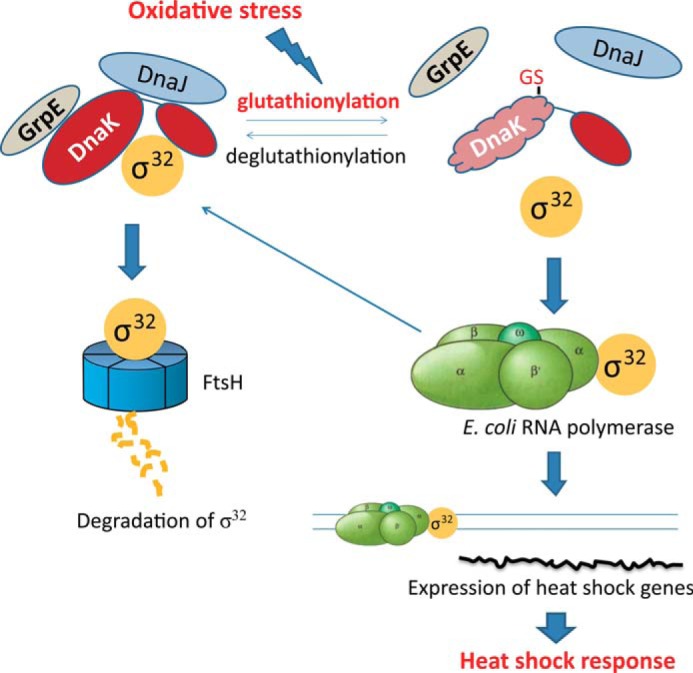
**Model illustrating how glutathionylation of DnaK provides a bridge between oxidative stress and the heat shock response.** Under non-stress conditions, DnaK binds to the heat shock transcription factor σ^32^ and targets it for degradation by the protease FtsH. Under stress conditions, σ^32^ is released from DnaK and induces transcription of heat shock-related genes. Under oxidative stress conditions, glutathionylation (*GS*) of DnaK may contribute to release of σ^32^.

Cys modification is suggested to enable Ssa1 to act as a redox sensor (72). Hsf1 is activated when cysteine residues in Ssa1 are mutated to asparagine to mimic oxidation of cysteine and are inactivated upon oxidative stress when cysteines are mutated to serine (72). Thus these results suggest a relationship among cysteine modification of Ssa1, activation of Hsf1, and the heat shock response. However, a mechanistic explanation is lacking for how interaction of Ssa1 and Hsf1 could be impaired by cysteine modification of Ssa1. It is widely accepted that oxidative stress can also trigger the heat shock response by activating Hsf1 in eukaryotes and σ^32^ in prokaryotes (31, 73). Hsf1 is inactivated by forming a complex with Hsp90, Hsp70, and Hsp40 under non-stress conditions (74–76), whereas σ^32^ is delivered by the DnaK-DnaJ-GrpE complex and degraded by the protease FtsH (23). Expression of inducible Hsp70 is autoregulated by interaction with Hsf1 or σ^32^ (74, 77). However, there is still no straightforward explanation of how Hsf1 or σ^32^ is released from Hsp70 upon stress. The most widely accepted hypothesis is that the large amount of nonnative proteins present under stress conditions competes with σ^32^/Hsf1 for binding to Hsp70 (57). Upon oxidative stress, which causes ATP depletion, glutathionylation of DnaK may occur, thus causing it to release σ^32^ instead of targeting it for degradation, which would then result in an increased half-life of σ^32^. This finding provides an alternative plausible explanation for how σ^32^ is activated at the onset of oxidative stress (Fig. 8). Although it now appears that both DnaK and Ssa1 can act as redox sensors by cysteine modification to regulate the heat shock response in bacteria and yeast, respectively, it remains to be confirmed whether such a mechanism is also found in higher eukaryotes. If so, the inducible forms of Hsp70 may represent a conserved redox sensor for activation of the heat shock response with cysteine modifications such as glutathionylation providing a bridge between oxidative stress and the heat shock response.

## Author Contributions

S. P. and C. C. conceived and supervised the study. H. Z. designed and performed the experiments and analyzed the data with assistance from J. Y. and input from S. W., W. G., C. C., and S. P. H. Z. and S. P. wrote the paper, and S. W., W. G., and C. C. revised it. All authors reviewed the results and approved the final version of the manuscript.

## Footnotes

4 H. Zhang, J. Yang, S. Wu, W. Gong, C. Chen, and S. Perrett, unpublished data.

## Footnotes

3 The abbreviations used are:

NBD: nucleotide-binding domain
SBD: substrate-binding domain
SEC: size exclusion chromatography
DTNB: 5,5′-dithiobis(2-nitrobenzoic acid)
LTQ: linear trap quadrupole
FP: fluorescence polarization.
